# Examining the effectiveness of Gateway—an out-of-court community-based intervention to reduce recidivism and improve the health and well-being of young adults committing low-level offences: study protocol for a randomised controlled trial

**DOI:** 10.1186/s13063-021-05905-2

**Published:** 2021-12-19

**Authors:** A. Cochrane, A. Booth, I. Walker, S. Morgan, A. Mitchell, M. Barlow-Pay, C. Hewitt, B. Taylor, C. Chapman, J. Raftery, J. Fleming, D. Torgerson, J. Parkes

**Affiliations:** 1 York Trials Unit, Department of Health Sciences, University of York, Heslington, York, YO10 5DD UK; 2 Primary Care, Population Sciences and Medical Education, University of Southampton, Southampton General Hospital, Southampton, SO16 6YD UK; 3 Hampshire Constabulary, Southampton Central Police Station, Southampton, SO15 1AN UK; 4 Department of Sociology, Social Policy and Criminology, University of Southampton, Southampton, SO17 1BJ UK

**Keywords:** RCT, Young adult offenders, Recidivism, Reoffending, Diversion, Police, Mental health, WEMWBS

## Abstract

**Background:**

Young adult offenders represent a third of the UK prison population and are at risk of poor health outcomes including drug and alcohol misuse, self-harm and suicide. Court diversion interventions aim to reduce the negative consequences of formal criminal justice sanctions and focus resources on addressing the root causes of offending. Although diversions are widely used, evidence of their effectiveness has not yet been established.

Hampshire Constabulary, working together with local charities, have developed the Gateway programme, an out-of-court intervention aimed at improving the life chances of young adults. Issued as a conditional caution, participants undertake a health and social care needs assessment, attend workshops encouraging analysis of own behaviour and its consequences and agree not to re-offend during the 16-week caution.

**Methods:**

This is a pragmatic, multi-site, parallel-group, superiority randomised controlled trial with a target sample size of 334. Participants are aged 18–24, reside in Hampshire and Isle of Wight and are being questioned for an eligible low-level offence. Police investigators offer potential participants a chance to receive the Gateway caution, and those interested are also invited to take part in the study. Police officers obtain Stage 1 consent and carry out an eligibility check, after which participants are randomised on a 1:1 basis either to receive Gateway or follow the usual process, such as court appearance or a different conditional caution. Researchers subsequently obtain Stage 2 consent and collect data at weeks 4 and 16, and 1 year post-randomisation. The primary outcome is the Warwick-Edinburgh Mental Well-being Scale (WEMWBS). Secondary outcomes include health status, alcohol and drug use, recidivism and resource use. The primary analysis will compare the WEMWBS score between the two groups at 12 months.

**Discussion:**

This pioneering trial aims to address the evidence gap surrounding diversion in 18–24-year-olds. The findings will inform law enforcement agencies, third sector organisations, policymakers and commissioners, as well as researchers working in related fields and with vulnerable target populations.

**Trial registration:**

International Standard Randomised Controlled Trial Register (ISRCTN 11888938).

## Administrative information

Note: the numbers in curly brackets in this protocol refer to SPIRIT checklist item numbers. The order of the items has been modified to group similar items (see http://www.equator-network.org/reporting-guidelines/spirit-2013-statement-defining-standard-protocol-items-for-clinical-trials/)

**Table:** 

Title {1}	Examining the effectiveness of Gateway – an out-of-court community-based intervention to reduce recidivism and improve the health and well-being of young adults committing low-level offences: study protocol for a randomised controlled trial
Trial registration {2a and 2b}.	The trial was prospectively registered with the International Standard Randomised Controlled Trial Register; ISRCTN Number: ISRCTN11888938.
Protocol version {3}	20^th^ May 2021, version 2.9
Funding {4}	The study is funded by the NIHR Public Health Research Programme, Ref 16/122/20. The intervention was funded by the Police and Crime Commissioner for Hampshire and Isle of Wight for the first year and Hampshire Constabulary thereafter.
Author details {5a}	Ann Cochrane^1^ Dr Alison Booth^1^ Dr Inna Walker^2^ Dr Sara Morgan^2^ Alex Mitchell^1^ Megan Barlow-Pay^2^ Professor Catherine Hewitt^1^ Inspector Ben Taylor^3^ Sergeant Caroline Chapman^3^ Professor James Raftery^2^ Professor Jenny Fleming^4^ Professor David Torgerson^1^ Professor Julie Parkes^2^ ^1^ York Trials Unit, Department of Health Sciences, University of York, Heslington, York, YO10 5DD ^2^ Primary Care, Population Sciences and Medical Education, University of Southampton, Southampton General Hospital, Southampton, SO16 6YD ^3^ Hampshire Constabulary, Southampton Central Police Station, Southampton, SO15 1AN ^4^ Department of Sociology, Social Policy and Criminology, University of Southampton, Southampton, SO17 1BJ
Name and contact information for the trial sponsor {5b}	Dr Alison Knight, Head of Research Integrity and Governance, Research and Innovation Services, University of Southampton, Southampton, SO17 1BJ
Role of sponsor {5c}	The study sponsor and funders have no input to the design or execution of the study or authority over subsequent publication of the findings.

## Introduction

### Background and rationale {6a}

Young adult offenders commonly have a range of health and social needs, making them vulnerable to mental health problems [1, 2]. Those aged between 18 and 24, who have been investigated for a suspected low-level offence, may need to attend court and, if convicted, face penalties such as prison. However, many believe that more should be done to prevent young adults from entering the criminal justice system to begin with. Diversion is a process whereby an accused offender is formally moved into a programme in the community, such as an out-of-court community-based intervention (OCBI), instead of entering the criminal justice system [3]. Despite the use of diversion programmes in the UK, particularly amongst a younger population [3–5], the evidence base around the effectiveness of diversion is still unclear.

The Gateway programme, an OCBI, has been developed by Hampshire Constabulary (HC), in partnership with local third sector organisations with the aim of improving the life chances of young adult offenders. In the programme, a mentor (Navigator) assesses the needs of each young adult and develops a pathway with referrals to healthcare and other local support services (e.g., housing). Young adults then take part in two workshops (LINX team) about empathy, and the causes and consequences of their behaviour. The components of the Gateway intervention are underpinned by theory and have been evaluated in isolation [6–13]; there has been no previous attempt to evaluate the combination of elements as used in the Gateway programme. This study therefore aims to assess the effectiveness of Gateway to improve the health and well-being of young adult offenders and reduce future criminal behaviour.

### Existing research

#### Diversion and recidivism amongst young populations

Literature searches were conducted using CINAHL, EMBASE, Europe PMC, MEDLINE, NIHR Library and Web of Science databases using the search terms: diversion, out of court disposals and court diversion. Studies on diversion have largely been undertaken outside the UK; the majority being conducted in the United States (US), with a few studies in Australia, New Zealand and the rest of Europe. Of the studies found, the majority focussed on younger populations and on family treatment as a therapeutic intervention. For example, multi-systemic therapy is a resource-intensive programme, which focuses on factors within the offender’s social network that contribute to their offending behaviour [14]. Treatment usually takes place within the community, such as at home or at school. A meta-analysis of diversion programmes for juvenile offenders was undertaken in 2012 and identified 28 studies involving 19,301 youths [15]. The most common outcome reported amongst the studies was recidivism, the tendency of the offender to reoffend. Of the five types of programmes included, a statistically significant reduction in recidivism was only observed for family treatment (OR = 0.57, 95%CI 0.40 to 0.82). Overall, there was high heterogeneity amongst the studies in terms of the research and programme design, as well as the quality of programme monitoring and implementation. The mean age of the population in studies identified by the meta-analysis ranged from 12.6 to 15.9 years of age. An evaluation of Checkpoint, a court diversion programme which does not respond to the needs of a particular age group but is aimed at adults, found a lower reoffending rate in comparison to the control cohort [16]. Despite the lack of robust evidence, the case for diversion amongst young adults is increasing, due to a growing recognition of their varying levels of maturity and complex needs [17, 18].

According to Hampshire Constabulary statistics for 2018/20, the five main offence categories for this age group where formal action was taken by the police are possession of drugs, violence, shoplifting, criminal damage and public order offences. These young adults often represent a vulnerable population with a range of complex needs, such as mental health issues and substance misuse. They are more likely to come into contact with the police both as suspects and victims of crime and are significantly over-represented in the formal justice process, accounting for approximately one third of police, probation and prison caseloads [18].

In the UK, a number of Police Forces are exploring the use of out-of-court disposals amongst 18–24-year-olds involved in less serious offending [19]. Out-of-court disposals are usually given where the offence is perceived to be a low-level crime. The aim is to divert the young adult away from their offending behaviour. However, evidence of the effectiveness of diversion interventions amongst this population remains limited.

#### Rationale for intervention and current study

The Gateway intervention model was conceived by HC as a ‘culture changing initiative’ that sought to address the complex needs of young adult offenders aged 18–24. Central to this is the belief that transitions into adulthood are not linear and that more work is necessary to support desistance amongst this vulnerable population. By combining components shown to have an impact, at least in the short term, the Gateway programme aims to provide a more comprehensive approach with longer-term impacts. HC understood the need to undertake a robust assessment of the effectiveness and cost-effectiveness of implementing the Gateway programme. Given the aims, the evaluation includes a wide set of outcomes in addition to reducing recidivism, with a particular focus on health and well-being of offenders and gives victim satisfaction.

### Objectives {7}

The aim of this RCT is to evaluate the effectiveness and cost-effectiveness of the Gateway programme issued as a conditional caution (intervention) compared to a court appearance or a different conditional caution (usual process).

The study objectives are to:

1. Examine the effectiveness of the Gateway intervention on (i) health and well-being including alcohol and substance use, (ii) access to and use of health and social services and (iii) quality of life, amongst young adult offenders
2. Explore the views and experiences of victims
3. Assess the quantity and quality of the Gateway intervention as delivered in the study and the generalisability of the findings
4. Identify and measure all relevant consequences, both cost and benefits, of the Gateway intervention compared with usual process
5. Examine the effectiveness of the Gateway intervention on reoffending

Objectives 1 and 5 will be addressed in the RCT; objectives 2 and 3 in the qualitative research and process evaluation and objective 4 in the economic evaluation.

### Trial design {8}

We are undertaking a pragmatic, superiority RCT with participants aged 18–24 who have committed low-level offences and reside within Hampshire and Isle of Wight, with an internal pilot phase. Participants will be randomised using a 1:1 allocation ratio to either the Gateway programme or disposal to a court summons or a different conditional caution to Gateway (usual process).

Economic and qualitative process evaluations will be undertaken in parallel with the RCT. This mixed methods approach will ensure the study evaluates the impact of the intervention on participants, the views of victims, assesses the intervention itself and examines the cost consequences of the Gateway programme.

## Methods: participants, interventions and outcomes

### Study setting {9}

The four trial recruitment sites are Southampton Central Police Station, Portsmouth Central Police Station, Newport Police Station on the Isle of Wight and Northern Hampshire Police Investigation Centre in Basingstoke, with recruitment from across the whole of Hampshire and Isle of Wight.

### Eligibility criteria {10}

The study population is 18–24-year-old offenders residing within Hampshire and Isle of Wight. According to police statistics, the five main offence categories for this age group are violence, possession or trafficking of drugs, theft, criminal damage and public order offences. These young adults represent a vulnerable population with a range of complex needs, such as mental health issues and drug and substance misuse. They are more likely to come into contact with the police both as suspects and victims of crime and are significantly over-represented in the formal justice process, accounting for approximately one third of police, probation and prison caseloads [18]. Consent and eligibility will include those dealt with in custody and disposed from police stations, and those dealt with through a voluntary interview and therefore disposed out of custody*.*

The following criteria need to be met for inclusion:

- Aged 18–24 years
- Lives within Hampshire and Isle of Wight where the Gateway programme is being delivered
- Anticipated guilty plea (i.e. admitted the offence and said nothing which could be used as a defence or has made no admission but has not denied the offence or otherwise indicated it will be contested)
- A disposal decision has been made (i.e. full code test has been met and public interest test met)

Potential participants will be excluded from the study if any of the following crimes have been committed and/or criteria apply:

- Hate crime according to Crown Prosecution Service (CPS) Policy
- Domestic violence-related crime
- Domestic violence-related crime referred to CPS
- Sexual offence as defined by the CPS
- Knife crimes—where a caution may be given if prosecution pursued
- Where on conviction the court is more likely to impose a custodial sentence (based on sentencing guides)
- A remand in custody order is sought
- Breach of court or sexual offences orders
- Any offence involving serious injury or death of another
- Any serious previous convictions within the last 2 years (i.e. serious violence, grievous bodily harm (GBH) or worse, serious sexual offences, robbery or indictable only offences)
- Summary offences more than 4 months old
- Persons subject to Court bail; Prison Recall, Red IOM (Integrated Offender Management) or currently under Probation
- Any indictable only offence
- All drink-drive or endorsable traffic offences (includes drug/driving)
- The offender already has a Gateway programme flag (cannot take part more than once)
- The offender needs an interpreter

### Who will take informed consent? {26a}

There are legal and practical issues precluding researchers from attending a Police station and custody areas to make direct contact with potential participants, before or after a disposal decision has been made. Often for those in custody, a disposal decision, including consent and randomisation, must be completed before they are released from custody. For logistical and safety reasons, a *two-stage* consent process is used in this study. Police investigators obtain Stage 1 consent, which includes (a) agreement to potential inclusion to the Gateway programme and (b) the optional sharing of their contact details for the University researchers to make direct contact. Potential participants are fully informed about the study prior to Stage 2 consent being obtained by the researchers at the first data collection time point, usually week 4 post-randomisation.

#### Stage 1 consent:

Police investigators dealing with potential participants receive training about the study, the consent process and use of the web-based eligibility and randomisation tool (Alchemer, formerly SurveyGizmo), to ensure standardised recruitment and recording of eligibility criteria.

**Fig. 1:**
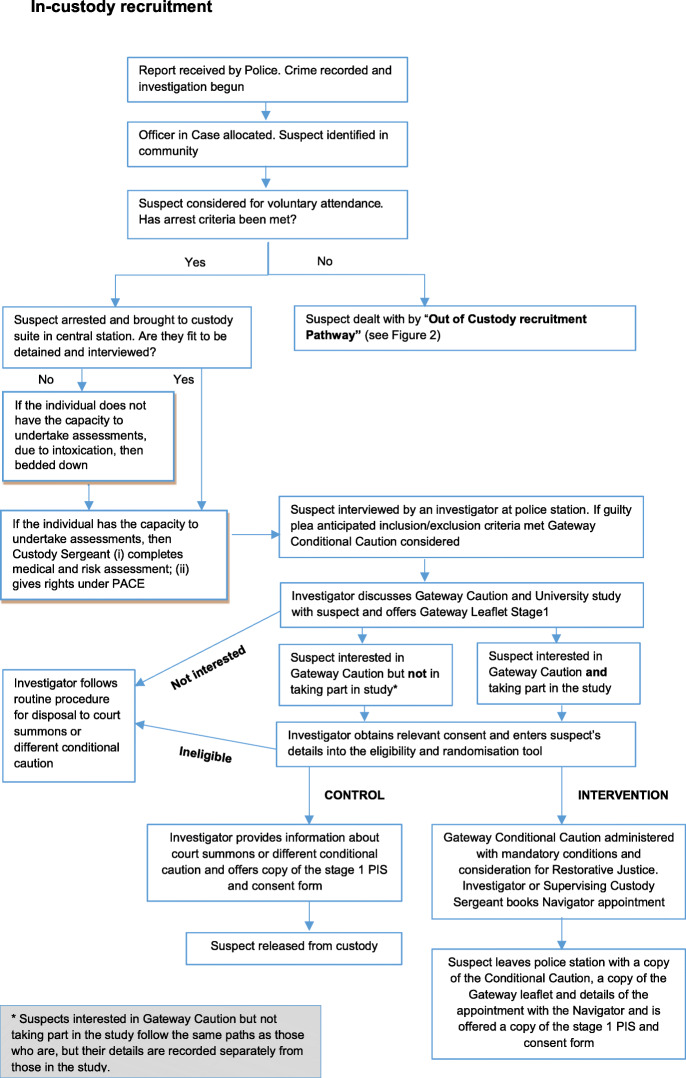
In-custody recruitment

During processing in custody (Fig. 1), investigators identify potentially eligible participants. For legal reasons, the investigator tells the individual about the potential option of a Gateway Conditional Caution as an alternative to prosecution or a different conditional caution. If the offender is interested in a possible Gateway caution, they are then informed about the ‘Questionnaire Study’ as it is referred to in participant facing materials. A Gateway Caution information leaflet (produced by HC independently of the study) and a *Questionnaire Study leaflet* may be offered or emailed later. Potential participants are made aware that further details about the study will be provided by a University researcher and that they may withdraw from the study at any time without giving a reason. If the offender agrees to take part in Gateway and the study, the investigator obtains their signatures on the combined Stage 1 Patient Information Sheet (PIS) and consent form.

Stage 1 consent includes permission for the Police to pass the individual’s contact details, such as *telephone number(s), email* and *postal addresses*, to the University researchers, and for the researchers to contact participants.

**Fig. 2:**
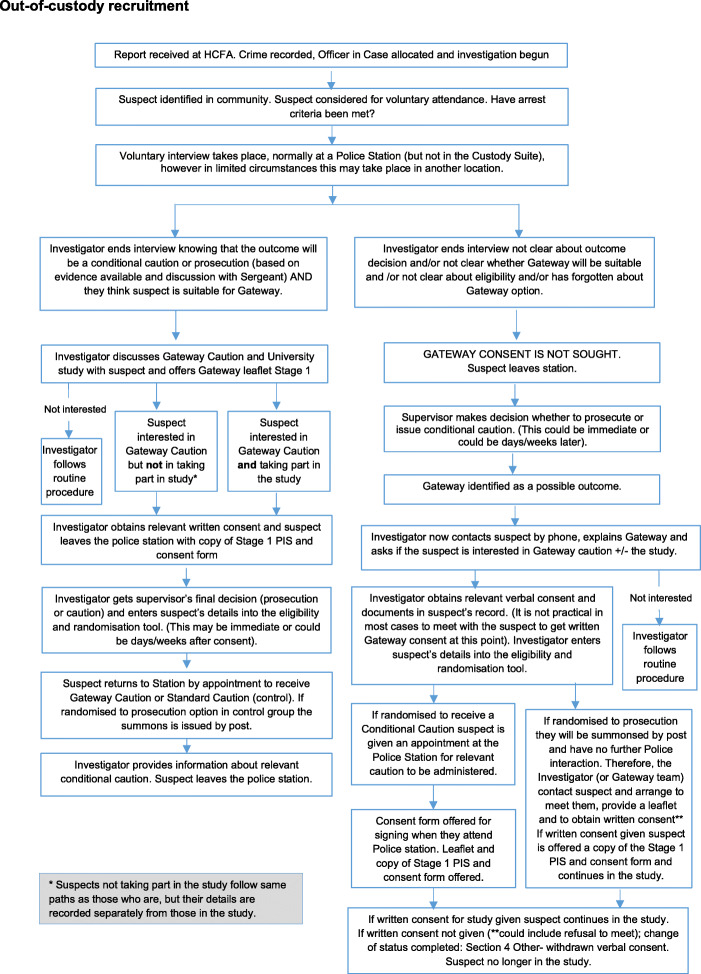
Out-of-custody recruitment

A small number of participants recruited through the out-of-custody process (Fig. 2) will be contacted by the Police, to have the Gateway caution option and study participation explained. If contacted by telephone, they will be asked to give verbal Stage 1 consent to participate. If given, this is recorded in the individual’s RMS incident record and their details entered in the eligibility tool and randomisation undertaken. Written consent is subsequently sought prior to any trial-related activities for the participant. Anyone later declining consent in writing is withdrawn from the trial. This approach ensures all potentially eligible participants have the chance to join the study and is in keeping with the pragmatic nature of this trial.

If the offender agrees to sharing their details with a researcher, the investigator obtains the offender’s written signature on the study consent page. The investigator will then enter the offender’s details in the web-based eligibility tool (Alchemer), and if eligibility is confirmed, randomisation will occur. The participant will be told their allocation at this point as part of their disposal.

#### Stage 2 consent

In advance of week 4 data collection, which is undertaken over the telephone, potential participants are first contacted by a researcher by telephone, and/or text, then by email and/or post if necessary. Once contact has been made, the Stage 2 PIS is emailed to the participant, or sent by post, if they have not already received a copy, and an interview date and time arranged. At the interview, or occasionally at the time of first contact, the researcher goes through the PIS providing explanations as required. Participants are provided with any other information they may need, and any queries answered. After time to consider their involvement, and if they decide to proceed, the researcher reads out the statements in the consent form. If the participant agrees to a statement, then the researcher puts the participant’s initials in the corresponding box. When complete, the researcher then adds their own name, the participant’s name and the date of verbal consent. The completed consent form is saved as a pdf and a copy is sent to the participant. Once consent has been given, data collection may occur at the same interview or on a subsequent day.

Participants are informed that they have the right to withhold consent or to withdraw from the study at any time without giving a reason. To maximise data collection, if a participant takes part in the week 16 interview having not taken part at week 4, verbal consent is obtained at that point.

### Additional consent provisions for collection and use of participant data and biological specimens {26b}

Stage 2 consent includes optional permission to access data from police records on recidivism for up to 10 years from their enrolment in the study. This is to facilitate a potential long-term assessment of re-offending as a separate follow-up at 10 years post-randomisation. This trial does not involve collecting biological specimens for storage.

## Interventions

### Explanation for the choice of comparators {6b}

The comparator for this RCT is a *usual process*. Under current guidance, for young adults aged 18–24, where there is enough evidence for a prosecution (known as full code test), it is considered in the public interest and where the individual admits responsibility for the offence, there are various possible outcomes. These include being given a conditional caution (but not a Gateway Caution), or referral for prosecution at court.

### Conditional caution

Conditions attached to conditional cautions must be appropriate, proportionate and achievable and have an element of rehabilitation and/or reparation and/or punishment [20]. Conditional cautions may have a mixture of conditions and the victim is consulted before the disposal decision is finalised. All conditions must be achievable and agreed by the offender. Examples of standard conditions include apology letters, victim awareness courses, drug diversion courses, alcohol diversion courses and fines or compensation. Drug, alcohol and victim awareness courses are provided through various organisations and the cost is charged to the offender. Conditions must be capable of being completed within 16 weeks, and in the event of non-compliance, the option of prosecuting the original offence is considered.

### Charge

This is an in-custody process. Where a young person is arrested and brought to custody, they will be interviewed by the investigating officer. If the evidence reaches the full code test and the offender is not suitable for a conditional caution, due to the nature of the offence or their previous convictions, the offender will be charged with the offence and given a court date before release from custody.

### Court summons

This is an out-of-custody process. If it is not necessary to arrest an offender, that is detain them in custody, then they are dealt with by way of voluntary interview. The offender can be interviewed under caution without arrest which means that they are free to leave at any time. When the investigating officer reaches the full code test, the file is submitted to the supervisor for a disposal decision. A summons is sent by post to the offender with a date to attend court.

### Intervention description {11a}

The Gateway programme is a police-led intervention delivered using a multi-agency approach. Participants randomised to the Gateway programme receive a 16-week conditional contract, known as a Gateway Conditional Caution. The conditions are to participate in the Gateway programme, participate in two LINX workshops and not re-offend during the length of the contract. A breach of the conditions may result in the offender being prosecuted for the original offence(s).

#### Content and delivery of Gateway intervention

##### Part 1: Initial assessment with a Navigator

Within 3–5 working days of their disposal, the Gateway Navigator will conduct a detailed needs assessment with the participant and mentor them through the programme. Based on the identified needs, the Navigator will connect the young adult with the appropriate services (e.g., alcohol, drugs, mental health and/or housing). The Gateway Navigators are trained support/case workers, provided by third sector organisation No Limits, and Southampton City Council. Assessments are held face to face whenever possible, but where necessary they are carried out via phone or video calls. Subsequent contacts are predominantly via telephone, text or video calling. Some additional face to face contact may take place based on risk factors and considering COVID-19 guidance and precautions. This would be in informal settings such as coffee shops or public outdoor locations.

##### Parts 2/3: The LINX workshops/telephone intervention

The LINX workshops aim to assist young adults in the development of cognitive and affective empathy, accept the need to change attitudes and behaviours including offending and prevent future anti-social and/or violent behaviour. LINX workshops for Gateway use carefully constructed experiential group work tools alongside a strong visual framework, ‘Making the LINX to rebuild my life’ wall, which represents the nine pathways to offending. LINX workshops should enable the young adult to explore and share personal feelings on a variety of issues, particularly around their life experience. The various exercises and activities throughout LINX workshops are designed to take the young adult on a journey; enabling them to see how an experience can create a feeling, which can be translated into a set of behaviours that, for these young adults, can create risk, and risk of offending.

##### Between weeks 2–3 post-randomisation

First workshop/telephone intervention uses materials designed to build and develop a relationship with the young adults’ personal Navigator. They in turn help the young adult identify risk factors leading to further offending. The first LINX workshop is delivered by the workshop leaders and is aimed at addressing: journey of offending; sentences and out of court disposals; empathy, rights, respect, and responsibility; impact of offending behaviour on victims/self and collateral damage to wider society; positive communication and relationship; restorative justice options and personal risk.

##### Between weeks 5–6 post-randomisation

Second workshop/telephone intervention is again broken down into sections and topics. The ‘Making the LINX to rebuild my life’ wall play a central part to the workshop. It assists in consolidating the learning and builds further on the young adults’ strengths. The young adults are helped to understand resilience and the part it plays in spinning life’s plates. Day 2 includes further examinations into personal risk and protective factors; the role self-esteem plays in keeping us and others safe; and identifying how positive communication can support our goals and make amends. The second day also assists the workshop leaders and Navigators in understanding if there are any gaps in support, whether new goals need to be set, and what they need to ‘keep their wall in order.’

The workshops ideally involve a group of individuals and take place face to face in a neutral venue. However, this is a pragmatic RCT and is being undertaken during the COVID-19 pandemic, which has necessitated changes to delivery of the intervention via telephone or video conferencing. This flexible approach reflects routine delivery of the intervention: where the number of attendees is insufficient to run a workshop, telephone/video conferencing may be used in order to fit delivery within the caution timeframe. The mode of delivery of all aspects of the intervention are recorded for the study and as far as possible will be accounted for in the analysis. Full details of the differences between the LINX workshops and the LINX telephone intervention have been documented and are available in the full protocol. As far as possible, it is only the mechanism of delivery that changes, the principle aims of the intervention and the content remain constant.

#### Provision of Gateway intervention

The Gateway Navigators are provided by No Limits, a third sector organisation that provides free advice, counselling, support and advocacy for under 26-year-olds, and by Southampton City Council. Agencies accessed following the triaging of needs include The Prince’s Trust, Two Saints (housing) and local Community Mental Health Teams.

The Hampton Trust are a third sector organisation, established in 1996 whose staff have extensive skills and expertise in developing community-based interventions for adults and young people including the LINX workshops, which they facilitate.

### Criteria for discontinuing or modifying allocated interventions {11b}

#### Custody sergeant prerogative

Duty Custody Sergeants approve all disposals and have the final say over whether someone can be randomised. They have all received training and understand the implications for the trial should they over-turn the disposal for a study participant.

#### Breach of conditions

Participants who are randomised to receive the intervention but who breach their Gateway Conditional Caution and are referred back for potential prosecution for their original offence will continue to be approached for data collection. When a caution is breached, discretion may be applied for the intervention and this involves use of a standardised decision flow chart. Where individual circumstances warrant it and discretion is applied, the individual is permitted to remain within the Gateway programme.

Similarly for control group participants who breach the conditions of a caution, standard police procedure is followed where discretion may be applied or the individual referred back for prosecution of their original offence.

#### Withdrawal from study (change of status)

Participants will be free to withdraw from the study at any point without giving a reason. Each PIS gives information on how a participant can withdraw, including who to contact. Forms for documenting type and reason of withdrawal and other applicable change of status categories are available for use by the Police Gateway Team and the University of Southampton (UoS) researchers.

Participants who withdraw from the study after giving Stage 1 consent but before giving Stage 2 consent will have their study details wholly anonymised by the University researchers where any personal details have been shared via Huddle. For analysis purposes, they will be continue to be treated as randomised to their allocated groups.

Participants who consent to Stage 1, but not to subsequent Stage 2 (without withdrawing), will not have any study assessments performed.

For participants who withdraw following Stage 2 consent, information already obtained up to that point will be retained. To safeguard the individual’s rights under UK GDPR only the minimum personally identifiable information will be retained by the Universities. Personal data will remain on Huddle, managed by HC, but no longer visible to the researchers and will not be downloaded or processed for the purposes of the study. Participant withdrawal after Stage 1 consent will require completion of a Change of Status CRF by the researchers.

Participants who decide to withdraw from the study at any stage will not undergo any further follow-up related to the study.

#### Loss of capacity during participation in the study

Should a participant lose mental capacity after consenting to take part, they will be withdrawn from the study.

### Strategies to improve adherence to interventions {11c}

Spreadsheets within Huddle provide the Police Gateway Team with oversight of the number of active clients in Gateway, numbers breached, number completed, indicators of breaches/completers, time to date in Gateway and time before breached, discretions applied, monthly recruitment and the numbers refusing participation and cases missed.

An ‘Engagement with client’ spreadsheet is maintained by Navigators to record adherence data and is available in Huddle. This spreadsheet includes participant ID; type of contact, date of contact, whether participant responded to contact, duration of contact in minutes, name of referring agency and comments from the Navigator. Third sector organisations liaise directly with the Police Gateway Team and Navigators to report engagement updates in accordance with referrals.

The Hampton Trust provide attendance registers for the LINX workshops/video/calls as these are mandatory sessions for the intervention.

Standard police monitoring of adherence to alternative conditional cautions issued to participants in the usual care group are followed and the information about the conditions and any breaches will be included in the results.

### Relevant concomitant care permitted or prohibited during the trial {11d}

There are no restrictions on concomitant care. For participants receiving the Gateway caution, an undertaking not to re-offend during the length of the caution is required. There are no other prohibitions related to the trial.

### Provisions for post-trial care {30}

There are no provisions within the study for post-trial care.

### Outcomes {12}

#### Primary outcome measure

The primary outcome measure is the Warwick-Edinburgh Mental Well-being Scale (WEMWBS) at 12 months. This is used to measure health and well-being amongst study participants. WEMWBS is a 14-item self-reported questionnaire that addresses mental health and well-being and has established valid reliable psychometric properties in adolescent populations [21, 22]. Compared to other well-being indices, WEMWBS was tested for response bias and showed low correlation with both subscales of the Balanced Inventory of Desirable Responding: Impression Management (*p* = 0.18*) and self-deception (*p* = 0.35**), which make it suitable for self-report [23]. Participants will self-report WEMWBS at 4 weeks, 16 weeks and 1 year post-randomisation (*p < 0.05, **p < 0.01).

#### Secondary outcome measures

The following are the secondary outcome measures:

- WEMWBS at 4 and 16 weeks post-randomisation.
- The SF-12 will be used to report health status. The 12 items of the SF-12 provide a representative sample of the content of the eight health concepts [24] and the various operational definitions of those concepts, including what respondents are able to do, how they feel and how they evaluate their health status.
- Risky alcohol use will be measured using the Alcohol Use Disorders Identification Test (AUDIT). The AUDIT tool is a simple screening tool that is used to identify the early signs of hazardous and harmful drinking and mild dependence. AUDIT has been validated amongst an adolescent [25, 26] and collage age population [27].
- Drug use will be measured using the Adolescent Drug Involvement Scale (ADIS). The ADIS was deemed most appropriate, as it captures recent/current use, and has been validated within this population age group [28].
- Reoffending type and frequency through access to routine data: police records will be used to examine the type and frequency of the trigger and subsequent offences up to 1-year post-randomisation.
- Data on resource use, including access to primary and secondary care health services and social care, will primarily be used to inform cost consequence analysis. Data will be obtained through self-reported responses to questions in the CRFs based on the customised Client Service Receipt Inventory [29].

### Participant timeline {13}

**Fig. 3:**
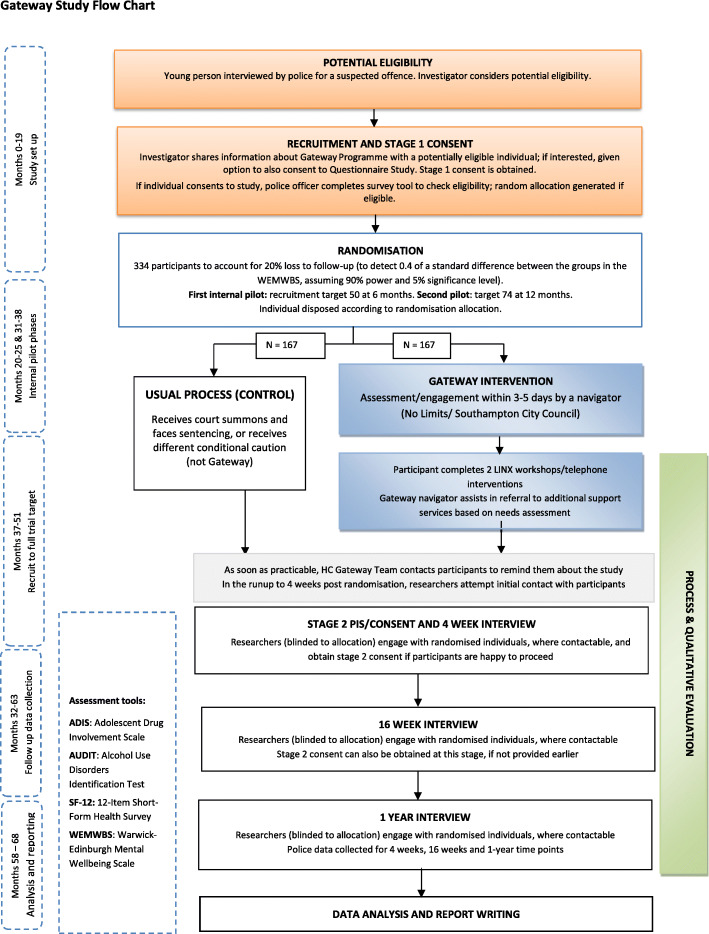
Gateway study flow chart

The time taken to complete investigations to initial discussion about entering the study at Stage 1 and then to disposal, when a participant has been randomised, can vary so timelines for this phase are necessarily flexible (Figs. 1, 2 and 3). The causes of delays in the process will be documented.

Participation in the intervention is for 16 weeks from the time of disposal.

Alternative conditional cautions issued to participants in the control arm will last a maximum of 16 weeks. For control arm participants allocated to a court summons, the timing will be variable, even more so as the pandemic has resulted in a backlog of cases. There will be more variability here such as the day the summons was raised, investigation completeness and if the disposal decision is made, in relation to the court date given.

For the study, all participants are asked to take part in telephone interviews at 4 weeks, 16 weeks and 1 year post-randomisation, with flexibility on timing anticipated to accommodate the target population. Participants missing one of the data collection points are followed up at the subsequent timepoint unless they have formally withdrawn from the study.

### Sample size {14}

There is no widely accepted and established minimal clinically significant difference for the primary outcome, WEMWBS. It has been suggested that a change of three or more points is likely to be important to individuals but different statistical approaches provide different estimates ranging from three to eight points (WEMWBS user guide [22]). There is also variation in the standard deviation of the WEMWBS with estimates ranging from 6 to 10.8 [30] with the pooled estimate of 10 across all studies. Assuming 90% power, 5% 2-sided statistical significance, mean difference of 4 points on WEMWBS and a standard deviation of 10, 266 participants are required. Preliminary figures from The Hampton Trust’s skills/attitudes workshops for domestic abuse (RADAR intervention) suggests a drop-out rate of approximately 15%. Conservatively, assuming a 20% attrition rate, 334 participants would need to be recruited and randomised.

### Recruitment {15}

The study population represents a vulnerable group with complex and overlapping health and social needs. They may include disadvantaged young adults, faced with previous and continuous adversity, such as unemployment, substance misuse and/or exposure to abuse. Our process for recruitment acknowledges that engaging with this target population is likely to be challenging.

The first 6 months of recruitment was run as an internal pilot to assess whether continued progression into the full trial was appropriate. According to police estimates at the start of recruitment, an average of 23 individuals would be eligible to receive the Gateway intervention each month once all the sites are recruiting.

Participants who consent at Stage 1 are now contacted by a member of the Police Gateway Team based at Southampton Central Police Station within a week of recruitment or as near to that time as practicable. The Police Gateway Team may attempt contacting the participants on more than one occasion and will also check if HC has received any updates on participants’ contact details should initial attempts be unsuccessful.

Participants are emailed a copy of the Questionnaire Study leaflet and a link to the Questionnaire Study video. The email also contains a study phone number and email address, to enable participants to contact the researchers directly.

The researchers attempt to contact participants in time to arrange the week 4 interview. The researchers provide participants with further information about the study and attempt to arrange the first telephone interview. A degree of flexibility is exercised, as dictated by the timing of the receipt of participants’ details following randomisation, and the availability of the participant and/or researchers.

The Police Gateway Team monitor recruitment on a daily basis, contact investigators where they see a case may be eligible to remind them about the Gateway caution and study, and discuss with investigators where a potential participant has been missed. All the Gateway and study documentation and information are readily available on the HC computer system. A variety of methods are used to raise and maintain awareness of the Gateway study within HC, such as computer screen savers, notices in police station offices and newsletter articles from the Deputy Chief Constable. Refresher training will be undertaken where necessary.

## Assignment of interventions: allocation

### Sequence generation {16a}

Participants will be allocated using simple randomisation with a 1:1 allocation ratio. The allocation sequence will be created using computer-generated random numbers in Alchemer using a randomisation sequence approved by the trial statistician. The system was tested and verified by York Trials Unit (YTU) data management and the trial statisticians during the training of police investigators, prior to the start of recruitment to the study.

### Concealment mechanism {16b}

Alchemer automatically generates and records the random allocation when a police investigator enters details for an eligible participant. It is not possible for investigators to predict or influence the allocation.

### Implementation {16c}

The allocation is generated when a police investigator enters the details of a potential participant, and they meet the eligibility criteria. The allocation of Gateway conditional caution or an alternative conditional caution or prosecution is displayed on the screen. The police investigator then informs the participant of the allocation and proceeds with disposal using the allocated intervention. A similar method for randomisation was adopted in an RCT of domestic abuse perpetrator intervention (CARA) conducted in Southampton Police District, where they were able to successfully recruit a similar population group (*n* = 293) [31].

## Assignment of interventions: blinding

### Who will be blinded {17a}

Consent for eligibility screening, Gateway consideration, sharing of contact details and the randomisation is undertaken by the police investigators, none of whom are involved in data collection for the study.

UoS Research team members involved in obtaining Stage 2 consent and data collection will be blinded as far as possible to the randomised group. The CRFs include a tick box for the researcher to indicate whether they believe blinding was compromised during assessment, or other communications such as when booking appointments, and if so which of the allocation groups they believe the participant to be in.

The statistician will not be blinded to treatment allocation.

### Procedure for unblinding if needed {17b}

No safety unblinding is foreseen in this study.

## Data collection and management

### Plans for assessment and collection of outcomes {18a}

Study outcome data will be recorded in paper Case Report Forms (CRFs) by researchers experienced in undertaking interviews. All three of the outcome data collection CRFs include WEMWBS, SF-12, AUDIT and ADIS measurement tools. Demographic data will also be collected in a CRF.

Following Stage 2 consent, the UoS researchers will read out the questions and answer options as set out in the relevant CRF. Guidance may be given if a question is not understood or requires further clarification. The researcher will hand-write the responses in the CRFs. Each CRF will be identified with a unique participant ID, signed and dated by the researcher and posted to YTU data management in a pre-paid envelope. The UoS researchers will track completion of each interview and CRF and YTU will track CRFs received and the date of receipt.

A participant may be reported as withdrawing from the study either by a researcher or a member of the Gateway police team using a Change of Status CRF. The Police Gateway Team may also report a participant withdrawing from the Gateway intervention.

A Case Management System (Huddle) is maintained centrally by Southampton Police as a key for linking the various sources of data for individuals together. For the purposes of analysis, data will be pseudonymised, and for subsequent reports and publications, the data will be wholly anonymised. For the purposes of ongoing data management, once randomised, individual participants will be identified using their unique study identification number including the site identifier they were recruited from.

Trial data and study files will be handled in accordance with Good Clinical Practice (GCP) principles, the appropriate data management procedures and YTU Standard Operating Procedures (SOPs).

### Plans to promote participant retention and complete follow-up {18b}

Early contact with participants by the Police Gateway Team and then the UoS researchers will be made to check that the telephone number given is active and to establish engagement.

Based on HRA guidance and advice from our Patient and Participant Involvement (PPI) representative, vouchers to the value of £30 for completion of week 4 CRFs, £40 for week 16 and £50 for a year 1 will be offered. A variety of methods to ensure the secure delivery of vouchers will be put in place to reduce selection bias, boost recruitment of those young adults with unstable living arrangements, without a bank account or lacking access to email or the Internet, who may otherwise not be inclined to participate.

### Data management {19}

#### Data entry and management

All staff involved in handling study data have been trained in data protection and data security. Trial data will be stored and transferred following YTU SOPs. Data will be processed according to trial-specific procedures and the Data Management Plan, all kept in the Trial Management File (TMF).

Paper CRFs received by YTU data management team will be scanned into OpenText Teleform, a secure, form processing software application that minimises the risk of data entry errors. Data queries will be raised with the UoS researchers and documented.

A Case Management System (Huddle) is maintained by the Police Gateway Team as a secure central location for data and document storage and sharing in line with the study Information Sharing Agreement. For the purposes of ongoing data management, once randomised, individual participants will be identified using their unique study identification number.

#### Data storage and archiving

Each site will hold data according to the UK General Data Protection Regulation (GDPR) and Data Protection Act (Great Britain 2018); data storage will be regularly reviewed to ensure compliance. Following Stage 2 consent, personal data and special category personal data will be processed in connection with this study under the legal basis of Article 6(1)(e) and Article 9(2)(j) of the UK General Data Protection Regulation (GDPR), for processing for the performance of a task carried out in the public interest, and as necessary for archiving purposes in the public interest, scientific or historical research purposes or statistical purposes, with Article 9(2)(j) operating in conjunction with the safeguard requirements set out in Article 89(1) of the GDPR.

All study files will be stored in accordance with GCP guidelines. Study documents (paper and electronic) will be retained in a secure (kept locked when not in use) location for the duration of the study. All essential documents, including source documents, will be retained for a minimum period of 10 years after study completion. The separate archival of electronic data will be performed at the end of the study, to safeguard the data for the period(s) established by relevant regulatory requirements. All work will be conducted following the University of York (UoY) Data Protection Policy [32].

### Confidentiality {27}

Participants will be assigned a unique identification number for use on consent forms, CRFs and other participant-specific records. Personal data will only be used when specific consent for its use for research purposes has been given to the investigator by the participants. Details will then be stored securely in the police research Case Management System (Huddle) for selective sharing with the research team only.

Electronic copies of Stage 1 and Stage 2 consent forms will be held securely in Huddle with limited and password-protected access. Personal information will not be released, except as necessary for the study and monitoring purposes.

All information provided for the purposes of this study will be kept strictly confidential. However, if a participant discloses information that may mean the future harm of another individual or relate to an offence for which they have not been charged, then the researchers are required to break that confidence by law. Examples of such disclosures are outlined in the Stage 2 PIS to make this risk clear to potential participants.

The research teams at the UoY and UoS will be the only individuals with access to study data. Primary data will be pseudonymised using the participant unique ID. A link back to the participant details will only be possible through the Document Management System (Huddle) and only UoS have access to shared personal contact details, in line with Stage 1 consent.

A police data analyst will ensure that all routinely collected police data is pseudonymised, using only the participant unique ID, before being transferred securely to the Universities. To access police data, a confidentiality agreement between the respective parties has been put in place.

## Statistical methods

### Statistical methods for primary and secondary outcomes {20a}

A detailed statistical analysis plan (SAP) will be agreed with the TMG and SSC/DMEC before all of the data have been collected. Amendments to the SAP will be agreed and clearly stated and justified. Analyses will be carried out using Stata v17 or later and follow the principles of intention-to-treat with outcomes analysed according to randomised group unless otherwise specified. Parameter estimates will be presented with associated 95% confidence intervals and *p* values, and significance testing will be two-sided at the 5% level unless otherwise stated.

The primary analysis will compare WEMWBS between treatment groups at 12 months and will be analysed using a covariance-pattern mixed-effect linear regression model, including assessments at 4 weeks, 16 weeks and 12 months post-randomisation, and treating participants as a random effect in order to account for repeated measurements amongst participants. Treatment group, time, group by time interaction, the total of the number of Record Management System (RMS) incidents and the number of Police National Computer (PNC) convictions 1 year pre-randomisation, age at randomisation, Index of Multiple Deprivation quintile at randomisation, pandemic time period and standard of usual process available will be adjusted for as fixed effects. If feasible, recruiting site will be included as a random effect. Pandemic time period refers to whether the follow-up took place before, during or after the COVID-19 pandemic, while standard of usual process available refers to the variation in the types of caution available during the pandemic. The inclusion of these two covariates reflects the guidance provided by Cro and colleagues for handling missing outcome data in randomised trials affected by the pandemic [33]. The treatment effect estimate at 12 months will be presented in the form of an adjusted mean difference, alongside the associated 95% confidence interval and *p* value. The treatment effect estimates at 4 weeks and 16 weeks will be presented in a similar manner as secondary outcomes.

The continuous secondary outcomes will be analysed in a similar manner to the primary outcome. Binary secondary outcomes will be analysed using a covariance-pattern mixed-effect logistic regression model and secondary outcomes classed as count data will be analysed using Poisson or negative binomial regression models. The models will adjust for the same fixed and random effects as the primary analysis.

### Interim analyses {21b}

There are no planned interim analyses for this study.

### Methods for additional analyses (e.g. subgroup analyses) {20b}

For the primary outcome, a subgroup analysis will be carried out comparing participants 18–21 years old to participants 22–24 years old (chosen on the basis that social services support care leavers up to 21 years old). The subgroup analysis will be carried out by repeating the primary analysis model with the addition of an interaction term between treatment group and subgroup.

### Methods in analysis to handle protocol non-adherence and any statistical methods to handle missing data {20c}

For the primary outcome, estimates of the effectiveness of the intervention under minimal and full compliance will be assessed using Complier-Average Causal Effect Analysis (CACE) [34]. The sensitivity of the primary analysis to the missing at random assumption will also be assessed.

### Plans to give access to the full protocol, participant-level data and statistical code {31c}

Reported here are the trial methods as set out in protocol version 2.9 20 May 2021. The full current protocol and previous versions showing protocol amendments to date are publicly available on the NIHR website: https://www.fundingawards.nihr.ac.uk/award/16/122/20.

Methods for the process, qualitative and economic evaluations are not reported here but are available in the full protocol.

Requests for other data or documentation should be made by contacting the corresponding author.

## Oversight and monitoring

### Composition of the coordinating centre and trial steering committee {5d}

The Trial Management Group (TMG) is the executive decision-making body and is responsible for overseeing the day-to-day running and management of the trial. The group comprises the Chief Investigator (CI and TMG Chair), Co-Investigators, Gateway Police Officers, Trial Manager, Qualitative & Process Evaluation Lead, Trial Statistician and Trial Coordinators. The TMG meets regularly with frequency according to the needs of the study. The CI has overall responsibility for the study. YTU is responsible for project management of the trial.

The SSC/DMEC, working to a study-specific Charter, provides overall supervision of the study on behalf of the sponsor and funders to ensure conduct is in accordance with the protocol. Membership of the SSC/DMEC comprises an independent Chair and three other independent members, a participant representative, the CI and the Senior Trial Statistician. Other study co-investigators may also attend the meeting at the invitation of the Chair. The committee meets at regular intervals and may request more frequent meetings when felt necessary.

The UoS is sponsor for this project and takes overall responsibility for the quality of study conduct.

### Composition of the data monitoring committee, its role and reporting structure {21a}

Due to the low-risk nature of the data in this study, the SSC will also take on the role normally undertaken separately by a data monitoring and ethics committee (DMEC). The independent members of the committee will be allowed to see unblinded data if required.

### Adverse event reporting and harms {22}

There are no anticipated adverse events or effects. The main risk for the participants is the nature of the topics discussed during outcome data collection interviews. These include discussions about their mental health, drug and substance use, and adverse childhood experiences. Offenders, who are allocated to receive the intervention, will also be encouraged to examine their own behaviour and its impact, which may be challenging and distressing for them. All assessments will be supported by the Navigators who are trained care workers experienced in dealing with this age group and discussing difficult issues.

The researchers and the care workers work closely and share experiences and concerns which can be raised at any of the relevant inter-disciplinary meetings.

### Frequency and plans for auditing trial conduct {23}

The study monitoring plan, approved by the funder and sponsor, is reviewed regularly. The low-risk nature of this study and COVID-19 restrictions mean this study is largely monitored centrally by delegated team members. There are no plans to externally audit the study, but this will be facilitated if the sponsor wishes.

### Plans for communicating important protocol amendments to relevant parties (e.g. trial participants, ethical committees) {25}

All protocol amendments are agreed by the TMG, and where appropriate, the SSC/DMEC and Funders prior to submission for research ethics and sponsor approval. Amendments are documented in the protocol and once approved, a copy sent to the funders, SSC/DMEC and the sponsor. Important protocol modifications will be submitted to *Trials* as an amendment or update to this journal article.

Police investigators involved in recruitment will be notified of any relevant protocol modifications via their internal project website and the regular newsletter issued by HC.

### Dissemination plans {31a}

The study has the potential to create a wide impact by influencing and improving health and welfare, public policy and public services, and culture and society. To maximise impact of the study, we have developed a strategy that engages with our project partners and the wider public, in particular our PPI representatives and PPP. Use will be made of existing networks and social media to share lay summaries of the findings. In addition, presentations will be given to the College of Policing and other relevant organisations, agencies and events.

The primary academic output from the study will be the report to the NIHR PHR Programme. In addition, peer review publications and conference presentations will include academic public health, criminology and policing audiences.

Development of an impact strategy and project plan for engagement with policy makers is being undertaken by the Public Policy Research Facility at the UoS. The findings of the study will directly inform police decision makers at operational and strategic level across Hampshire and in other police forces. The study also has the potential to inform decisions at the level of the Crown Prosecution Service, Government legislators, where community outcomes like Gateway are part of the suite of mandated outcomes for offences.

## Discussion

The design of this novel study has evolved in response to real-world challenges requiring pragmatic adaptions. These have included revision of recruitment eligibility and increasing site participation; tailoring consent processes to accommodate the legal basis of police processes in line with UK GDPR regulations and changing the mode of contacting and engaging with our hard-to-reach study population.

In addition to living through the current pandemic, the changes in restrictions also impacted police practice. We have responded positively to each of these as appropriate, often with exploratory mitigating strategies. Barriers to participation will continue to be addressed, particularly for those participants in education, who work and/or with childcare commitments, are unemployed or are experiencing mental health difficulties. The above scenarios have been encountered in the pilot. Our PPI representatives have provided valuable advice in addressing many issues already and we will continue to seek their advice on mitigating barriers.

Lockdown measures prompted a change from initial face to face meetings with participants to engagement by telephone. Responses so far suggest a preference for over-the-phone interviews. Gathering of data with consent across both groups has not been done before using this methodology in this age group, with a non-recidivism primary endpoint.

The research processes have been successfully integrated with police usual processes. Challenges still arise; however, the increased level of communication and commitment by our co-production approach already demonstrates potential for future collaborations.

The pandemic has been a major driver for the current design of this pragmatic trial and the recruitment and retention strategies adopted. These have enabled recruitment and data collection during lockdowns and social distancing, future proofing for the duration of the remaining recruitment period during continued uncertain times.

Multi-agency collaboration remains at the heart of the Gateway programme and delivery of this RCT. This unique study is expected to contribute robust evidence to allow local and national Police Commissioners to evaluate the possible benefits not just in the primary outcome of mental health, but on recidivism. The results will be used to inform policy decisions and best practice for similar interventions across the UK.

## Trial status

The current UoS REC-approved version of the protocol is version 2.9, 20 May 2021. This manuscript is a restructured and edited version of the current protocol to comply with the SPIRIT guidelines. Recruitment into the Gateway trial commenced in 1 October 2019 and the internal pilot phase was successfully completed. National restrictions in response to the COVID-19 pandemic resulted in the temporary suspension of recruitment from 22 March 2020 to 4 September 2020. Recruitment is ongoing at the time of manuscript submission, and to date, 163/334 participants have been randomised (2021). As a result of delays in study setup and lower than anticipated recruitment rates, a request for a funded extension has been submitted to NIHR PHR with an anticipated recruitment completion date of September 2023.

## Abbreviations

ACE: Adverse Childhood Experiences questionnaire
ADIS: Adolescent Drug Involvement Scale
AUDIT: Alcohol Use Disorders Identification Test
BRFSS: Behavioural Risk Factor Surveillance System
CARA: Conditional Cautioning and Relationship Abuse
CI: Chief Investigator
CPS: Crown Prosecution Services
CRF: Case Report Form
CSRI: Client Service Receipt Inventory
DMEC: Data Monitoring and Ethics Committee
HC: Hampshire Constabulary
HMPPS: Her Majesty Prison and Probation Services
HRA: Health Research Authority
HYOT: Hampshire Youth Offenders Team
IoW: Isle of Wight
NHS: National Health Service
NOMS: National Offender Management Services
OCBI: Out-of-court community-based intervention
OR: Odds ratio
PI: Principal Investigator
PIS: Participant Information Sheet
PNC: Police National Computer
PPI: Patient and Public Involvement
PPP: Public Participation Panel
REC: Research Ethics Committee
RCT: Randomised controlled trial
RJ: Restorative justice
RMS: Record Management System
SF-12: Short Form 12 questionnaire
SSC: Study Steering Committee
TMG: Trial Management Group
UoS: University of Southampton
UoY: University of York
WEMWBS: Warwick-Edinburgh Mental Well-being Scale
YTU: York Trials Unit
